# Preparatory Course

**Published:** 1859-11

**Authors:** 


**Preparatory Course.**

The Preparatory or Elementary Course of the Atlanta Medical College opened on the first Monday in November, with a most encouraging class. This is a most valuable addition to the course of instruction, affording a most profitable substitute for the old plan of office study, which is a perfect farce. We would again repeat that this course imposes no additional expense for fees, the amount paid being deducted from the fees of the regular course.

